# In Vivo Investigation of 3D-Printed Calcium Magnesium Phosphate Wedges in Partial Load Defects

**DOI:** 10.3390/ma17092136

**Published:** 2024-05-02

**Authors:** Elke Hemmerlein, Elke Vorndran, Anna-Maria Schmitt, Franziska Feichtner, Anja-Christina Waselau, Andrea Meyer-Lindenberg

**Affiliations:** 1 Clinic for Small Animal Surgery and Reproduction, Ludwig Maximilians University Munich, 80539 Munich, Germanya.waselau@lmu.de (A.-C.W.); ameylin@lmu.de (A.M.-L.); 2 Department for Functional Materials in Medicine and Dentistry, University of Würzburg, 97070 Würzburg, Germanyanna-maria.schmitt@meduniwien.ac.at (A.-M.S.)

**Keywords:** biocompatibility, material degradation, bone cements, calcium magnesium phosphate cements, 3D powder printing

## Abstract

Bone substitutes are ideally biocompatible, osteoconductive, degradable and defect-specific and provide mechanical stability. Magnesium phosphate cements (MPCs) offer high initial stability and faster degradation compared to the well-researched calcium phosphate cements (CPCs). Calcium magnesium phosphate cements (CMPCs) should combine the properties of both and have so far shown promising results. The present study aimed to investigate and compare the degradation and osseointegration behavior of 3D powder-printed wedges of CMPC and MPC in vivo. The wedges were post-treated with phosphoric acid (CMPC) and diammonium hydrogen phosphate (MPC) and implanted in a partially loaded defect model in the proximal rabbit tibia. The evaluation included clinical, in vivo µ-CT and X-ray examinations, histology, energy dispersive X-ray analysis (EDX) and scanning electron microscopy (SEM) for up to 30 weeks. SEM analysis revealed a zone of unreacted material in the MPC, indicating the need to optimize the manufacturing and post-treatment process. However, all materials showed excellent biocompatibility and mechanical stability. After 24 weeks, they were almost completely degraded. The slower degradation rate of the CMPC corresponded more favorably to the bone growth rate compared to the MPC. Due to the promising results of the CMPC in this study, it should be further investigated, for example in defect models with higher load.

## 1. Introduction

The treatment of larger, complex bone defects with bone substitutes is still a major challenge. Autografts and allografts are considered the gold standard for filling defects. However, these have certain disadvantages. The use of autografts is limited by complications such as donor-site pain or morbidity, prolonged operation time, increased blood loss and scarring. Also, there is only a limited amount that can be harvested [1,2,3]. Allografts are the most commonly used alternative to autografts, but they carry the risk of transmitting infection, graft fatigue fracture, non-union or immune rejection [2,3]. Synthetic bone graft substitutes have been developed to overcome these limitations. They offer unlimited availability, are inexpensive and can be produced individually and for specific defects due to their adjustable parameters such as shape, porosity or composition [1]. Additionally, they have a long shelf life, and the risk of disease transmission is avoided [1,3].

The ideal bone substitute is biocompatible, osteoconductive and degradable, being replaced by new bone tissue and ensuring mechanical stability at the same time [4]. The optimum degradation time for bone substitutes should coincide with the bone regeneration process. This requires the bone substitute to maintain its mechanical integrity and provide stability, especially during the early stages of bone healing. As bone tissue regenerates, the bone substitute should gradually degrade to be replaced by newly formed bone [5]. The degradation rate is influenced by factors such as composition, shape and porosity of the bone substitute. Bone regeneration depends on several variables including the age and health of the individual, the implantation site, and the defect size [6,7,8]. Among the most commonly used clinical bone substitutes are calcium phosphate cements (CPCs), such as hydroxyapatite (HA) and tricalcium phosphate (TCP), as they resemble the mineral phase of bone and are therefore characterized by excellent biocompatibility [9]. However, depending on the material, they very slowly or even hardly degrade in vivo and have so far only been used for non-load-bearing defects, as they are brittle and have low mechanical stability [10].

Magnesium phosphate cements (MPCs) have been increasingly researched in recent years as a possible alternative to CPCs [11]. These are characterized by a higher initial strength due to the magnesium substitution and degrade faster than pure CPCs due to their higher in vivo solubility. Magnesium (Mg) also has a favorable influence on bone metabolism [12,13]. The positive effect of released Mg ions on bone remodeling results in faster ingrowth of bone tissue, which has already been shown with scaffolds of different Mg alloys [14,15] and is also assumed for Mg-based cements [13,16]. Furthermore, in an in vitro study by Ostrowski et al. [17] in which amorphous (ATMP) and crystalline (CTMP) trimagnesium phosphate pellets were examined, it was described that Mg ions induce the mineralization of an amorphous, HA-like phosphate phase on the scaffold surface of ATMPs, which has a stimulating effect on the proliferation and activity of osteoblasts. However, the differentiation of monocytes into osteoclasts is inhibited, which leads to reduced bone resorption. In another in vitro study by Roy and Bose [18], Mg-doped β-TCP was investigated, and it was found that Mg significantly slows down osteoclastogenesis. MPCs have already been successfully tested in the form of cement pastes (struvite and K-struvite) in partially loaded large animal models [19,20].

Calcium magnesium phosphate cements (CMPCs) appear to have better biological properties than pure CPCs and MPCs due to a more favorable combination of mechanical stability and biocompatibility [11]. The magnesium component increases the chemical solubility, degradation rate, resorption and osteoconductivity [21,22]. So far, however, there is little research on the in vivo investigation of CMPCs. They have already been investigated in smaller, unloaded defect models, in which excellent biocompatibility, rapid degradation behavior and good osseointegration were observed [21,23,24].

CMPCs have been used in previous in vivo studies, including in the form of self-setting pastes [25], granules [16] and 3D powder-printed cylinders [23]. The advantage of 3D printing compared to conventional ceramic processing methods is that patient-specific scaffolds are produced based on computed tomography data, which is particularly beneficial for large and complex defects [26,27]. By adjusting the external structure and porosity, biological and mechanical properties can be adapted to the tissue being replaced [27]. Three-dimensional powder-printed scaffolds can be produced inexpensively and comparatively quickly and can be used immediately without waiting for the scaffold to set [28,29]. In addition, 3D powder-printed scaffolds offer a high microporosity that promotes the ingrowth of vessels, cells and nutrients by facilitating diffusion into the scaffold, which has a positive effect on osteogenesis [27,30]. The production method is suitable for processing bone substitutes based on CPCs, MPCs or CMPCs, which have shown excellent biocompatibility, favorable degradation behavior and osteoconductivity both in vitro [27,29,31] and in vivo [23,24].

CMPCs have so far only been investigated in unloaded defect models and showed excellent results in terms of biocompatibility, degradation and osseointegration. Their behavior in partially loaded bone is not yet known, which is why the aim of the present study was to investigate the degradation and osseointegration behavior and the stability of CMPCs compared to MPCs in a larger defect. For this purpose, wedge-shaped MPC and CMPC scaffolds were fabricated using 3D powder printing techniques and examined for the first time in a partially loaded, segmental defect model of the rabbit tibia in vivo over a period of 24 and 30 weeks.

## 2. Materials and Methods

### 2.1. Production and Characterization of the Scaffolds

#### 2.1.1. Production of the Scaffolds

Due to excellent results, the production of the wedges is based on the studies by Kowalewicz et al. [23,24]. The 3D powder printing and post-treatment process followed a procedure similar to that of the study by Schaufler et al. [32]. By sintering (at 1100 °C for 5 h each) mixtures of calcium hydrogen phosphate (CaHPO_4_, J.T. Baker, Phillipsburg, NJ, USA), calcium carbonate (CaCO_3_, Merck, Darmstadt, Germany), magnesium hydrogen phosphate (MgHPO_4_·3H_2_O, Alfa Aesar, Kandel, Germany) and magnesium hydroxide (Mg(OH)_2_, VWR International GmbH, Darmstadt, Germany), cement powders of the general chemical composition Ca_x_Mg_3−x_(PO_4_)_2_ with x = 0 (MPC) and 0.75 (CMPC) were synthesized in corresponding molar ratios (Table 1).

**Figure 1:**
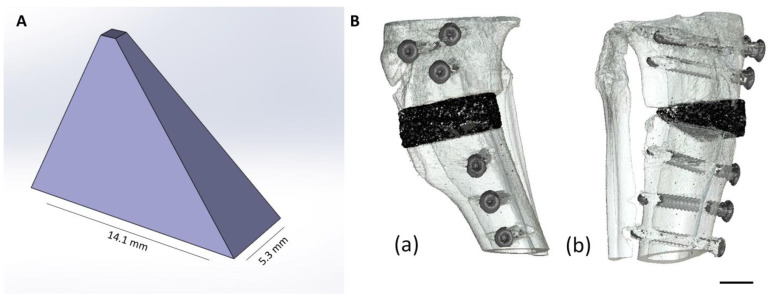
(**A**) A model of the wedge (SolidWorks, Dassault Systèmes SolidWorks Corp., Waltham, MA, USA): oblique view. (**B**) 3D reconstruction of the wedge (black) in the proximal rabbit tibia: (**a**) medial view; (**b**) craniolateral view. Scale bar = 5 mm.

The sinter cakes were then crushed using a mortar and pestle. In total, 125 g of each cement fragment was ground in a planetary ball mill (PM400 Retsch, Haan, Germany) in 500 mL zirconia beakers, each with 4 zirconia balls (Ø = 30 mm), at 200 rpm for 10 min. The ground cement powder was then sieved to a particle size <355 µm. For powder printing, the cement powder was homogeneously mixed with 4 wt% hydroxypropyl methylcellulose (Sigma-Aldrich, Steinheim, Germany) for 20 min in a plowshare mixer. The cement powders modified with cellulose served as the powder phase for 3D powder printing. Wedge-shaped scaffolds (*n* = 42) were produced using the 3D powder printer from ZCorp (Z310, Z-Corporation, Burlington, VT, USA). The base area of the wedge was 14.1 × 5.3 mm (l × w), with a height = 10.4 mm (Figure 1A).

Distilled degassed water was used as the printing solution. A layer thickness of 100 µm and a binder/volume ratio of 0.275 were selected for printing. The scaffolds were removed from the powder bed after a drying time of 1 h (room temperature) and dedusted using compressed air. The organic phase was removed at 500 °C for 2 h. This was followed by a further sintering phase of 4 h with a phase-dependent final sintering temperature. In the case of Ca_0.75_Mg_2.25_(PO_4_)_2_, sintering in the final phase took place at 1150 °C, while Mg_3_(PO_4_)_2_ was sintered at 1200 °C. After the sintering process, scaffolds made of Ca_0.75_Mg_2.25_(PO_4_)_2_ were infiltrated four times with a 2 M H_3_PO_4_ solution (phosphoric acid) (1st infiltration: 150 µL, 2nd infiltration: 90 µL, 3rd infiltration: 65 µL, and 4th infiltration: 40 µL) so that the pore volume was completely filled. After each infiltration, the wedges were dried for 24 h at room temperature (RT). Scaffolds based on Mg_3_(PO_4_)_2_ were aged for 24 h in a 3.5 M (NH_4_)_2_HPO_4_ (diammonium hydrogen phosphate, DAHP) (Merck, Darmstadt, Germany) solution. The wedges were then air-dried at room temperature. Before implantation, all scaffolds were washed to achieve a neutral pH value. For this purpose, the wedges were placed in a Petri dish with a washing solution (3 mL of water/scaffold for 1 h, then phosphate-buffered saline (PBS; 8.0 g NaCl, 0.2 g KH_2_PO_4_, 1.1 g Na_2_HPO_4_ and 0.2 g KCl in 1 l H_2_O) for 15 min) and stored on a rocker table. After drying at room temperature, the scaffolds were individually wrapped and γ-sterilized with >25 kGy radiation (BBF Sterilization Service GmbH, Kernen, Germany).

#### 2.1.2. Chemical Composition

The chemical composition of the scaffolds was determined using X-ray diffractometry and Rietveld analysis. To determine the qualitative phase composition, three wedges of each composition were finely ground and placed in a cuvette of the diffractometer (D8 Advance, Bruker Corporations, Karlsruhe, Germany). The measurements were carried out under the following conditions: Cu-Kα radiation, measurement angle 2θ = 10–40°, measurement speed of 0.5 s/step and rotation of the cuvette of 15 rpm.

The phase composition was determined using DIFFRAC.EVA V.5.1.0.5. (Bruker Corporations, Billerica, MA, USA) based on ICDD reference patterns: MgHPO_4_·3H_2_O (newberyite; PDF Ref. 00-020-0153), CaHPO_4_·2H_2_O (brushite; PDF Ref. 00-009-0077), NH_4_MgPO_4_·6H_2_O (struvite; PDF Ref. 00-015-0762), Mg_3_(PO_4_)_2_ (farringtonite; PDF Ref. 00-033-0876), Ca_4_Mg_5_(PO_4_)_6_ (stanfieldite; PDF Ref. 00-011-0231) and MgO (magnesium oxide/periclase; PDF Ref. 00-004-0829). A quantitative phase analysis was carried out using the Rietveld method. The TOPAS V6 software (Bruker Corporations, Billerica, MA, USA) was used for this purpose.

#### 2.1.3. Determination of Compressive Strength

The compressive strength was determined on 10 rectangular specimens (l = 6 mm, w = 6 mm, h = 12 mm), which were previously aged for 24 h at 37 °C in PBS. A testing machine (Z010, Zwick GmbH, Ulm, Germany) with a 10 kN load cell, a preload of 1 N and a test speed of 1 mm min^−1^ were used for the measurement. Compressive strength testing was performed according to ISO 13175-3 2012 [33].

#### 2.1.4. Porosity

For the porosity measurements, the wedges were broken into two parts and placed in the measuring cuvette of the porosimeter (Pascal 140/440, Thermo Fisher Scientific Inc., Waltham, MA, USA) to record the complete porosity of a scaffold. The measurements were carried out on three wedges for each material variant in a pressure range of 0.01 kPa–400 MPa. The SOLID software (SOLver of Intrusion Data Ver. 1.6.5, Thermo Fisher Scientific Inc. Waltham, MA, USA) was used to analyze the data.

#### 2.1.5. Energy Dispersive X-ray Analysis (EDX) and Scanning Electron Microscopy (SEM) before Implantation

The scaffold wedges were fixed in 4% formalin solution and then dehydrated in an ascending alcohol series. After degreasing in xylene (Carl Roth GmbH, Karlsruhe, Germany), they were embedded in a plastic embedding system based on methyl methacrylate (Technovit^®^ 9100, Heraeus Kulzer GmbH, Wehrheim, Germany). Using the cutting–grinding technique according to Donath [34], 50–80 µm thick sections were produced with a diamond band saw and a grinding machine (Cut-Grinder and Lap-Grinder, Messner GmbH, Oststeinbek, Germany). One unstained thick section per material and time group was examined using energy dispersive X-ray spectroscopy (EDX) and scanning electron microscopy (SEM). Before they could be examined by means of field emission electron microscopy (Crossbeam CB 340, Zeiss, Oberkochen, Germany), the thick sections were coated with platinum (4 nm thickness) using a sputter coater (Leica EM ACE600, Leica Mikrosysteme GmbH, Wetzlar, Germany). EDX images were obtained using a system with a silicon drift detector (INCA Energy 350 AzTec Advanced system with silicon drift detector) from Oxford Instruments (Abingdon, UK). The scaffold center was analyzed with an accelerating voltage of 10 keV at a magnification of 28× and 500×. The scaffolds were assessed in the SEM based on the surface structure and pore composition. EDX was used to examine the phase distribution based on the presence of magnesium (Mg), calcium (Ca) and phosphate (P) ions.

### 2.2. Animal Model

The animal experiment was approved by the Government of Upper Bavaria according to paragraph 8 of the German Animal Welfare Act (approval number Az. ROB 55.2-2532.Vet_02-19-64). For the present study, 42 adult female Zimmermann rabbits (ZiKa rabbits, Asamhof Kissing, Germany) with an age of 6 months and with an average weight of 4.5 kg were used. The animals were divided into four time groups (6, 12, 24 and 30 weeks post-operation). Six rabbits per time group were implanted with the materials CMPC and MPC (Figure 1B). In the 30-week group (plate removal group, PR), six rabbits had CMPC wedges implanted, and the PEEK plate used for fixation was removed after 24 weeks. The animals were kept in conventional cages (Scanbur A/S, Karlslunde, Denmark) and received daily rationed pellet feed (Kanin Kombi, Rieder Asamhof GmbH, Kissing, Germany) as well as hay and water ad libitum.

#### 2.2.1. Implantation of the Wedges

The surgical method was analogous to the study by Schmidt et al. [35] and was based on a high tibial open-wedge osteotomy [36].

Before surgery, the analgesic meloxicam (0.3 mg/kg, Meloxoral^®^ 1.5 mg/mL, Dechra Veterinary Products Deutschland GmbH, Aulendorf, Germany) and the antibiotic enrofloxacin (10 mg/kg, Orniflox^®^ 25 mg/mL, Dechra Veterinary Products Deutschland GmbH, Aulendorf, Germany) were administered orally. Ketamine (15 mg/kg, Anesketin^®^ 100 mg/mL, Dechra Veterinary Products Deutschland GmbH, Aulendorf, Germany) and medetomidine (0.25 mg/kg, Dorbene vet^®^ 1 mg/mL, Zoetis Deutschland GmbH, Berlin, Germany) were then injected intramuscularly to induce anesthesia. After securing the airway by means of endotracheal intubation, anesthesia was maintained using isoflurane (1.5–2.0 vol%) with a simultaneous supply of oxygen (1 L/min). For analgesic coverage, the animals also received fentanyl 1 μg/kg/h (Fentadon^®^ 50 µg/mL, Dechra Veterinary Products Deutschland GmbH, Aulendorf, Germany) as a continuous drip infusion. After aseptic preparation of the surgical field on the right hind limb, access was made with an approximately 3 cm long skin incision on the medial side of the proximal tibia. The surrounding muscles and periosteum were then dissected using a raspatory. Using a saw (Colibri II, DePuy Synthes^®^, Synthes GmbH, Oberdorf, Switzerland), a wedge-shaped defect corresponding to the size of the scaffold was created in the proximal tibia while protecting the fibula. The scaffold wedge was inserted into the defect to fit precisely against the cortical bone. The scaffold was then fixed using a T-plate (PEEK, RISystem AG, Landquart, Switzerland) made of non-resorbable plastic and three titanium screws (Cortex Screw Stardrive Ø 1.5 mm, DePuy Synthes^®^, Synthes GmbH, Oberdorf, Switzerland) proximal and distal to the defect. Subsequently, after adaptation of the fascia, the subcutis was closed using continuous sutures (Monosyn violet 4/0, B. Braun Melsungen AG, Melsungen, Germany) and the skin was closed using single-button sutures (Optilene blue 4/0 B. Braun Melsungen AG, Melsungen, Germany). Following the operation, X-rays in two planes and µCT examinations of the surgical area were performed. Atipamezole (25 mg/kg, Atipam^®^, Albrecht GmbH, Aulendorf, Germany) was then injected intramuscularly to antagonize medetomidine. As post-operative analgesia, the rabbits received 20 µg/kg buprenorphine (Buprenovet^®^ Multidose, 0.3 mg/mL, Bayer Vital GmbH, Leverkusen, Germany) up to three times daily for four days and 0.3 mg/kg/day meloxicam until the fifth day after surgery. For antibiotic coverage, the animals were also administered 10 mg/kg/day enrofloxacin until post-operative day 5. If necessary, the animals were also given the analgesic metamizole at 40 mg/kg (Novaminsulfon—1A Pharma^®^, 500 mg/mL, 1A Pharma GmbH, Oberhaching, Germany) two to three times a day. The rabbits were clinically examined daily for pain, lameness and wound control until day 14 post-surgery.

#### 2.2.2. Plate Removal

To investigate bone remodeling after defect healing, the PEEK plate used for fixation was removed from six animals in the CMPC group (30-week group) at 24 weeks post-operation, after prior verification of cortical bone healing using X-ray examinations. Preparation of the surgical field, anesthesia, analgesia and post-operative care were performed in the same way as for the implantation surgery. Subsequently, X-ray and µ-CT examinations were also performed at 26, 28 and 30 weeks post-plate removal surgery.

### 2.3. Radiological Examination and Semi-Quantitative Evaluation

**Figure 2:**
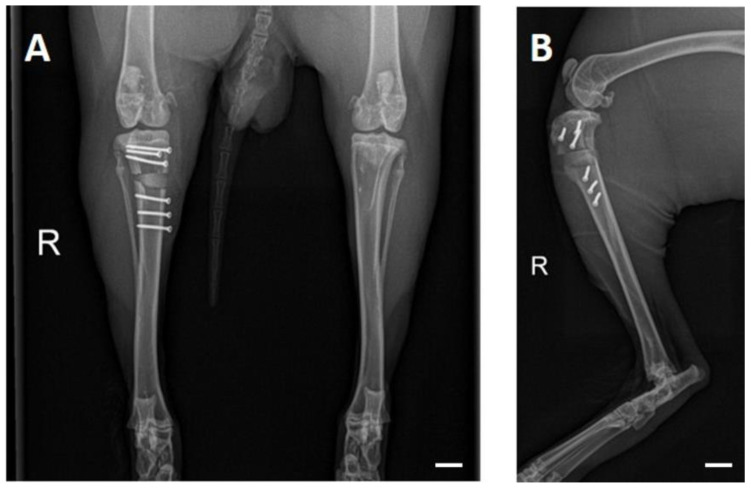
Post-surgery radiographs: (**A**) craniocaudal and (**B**) mediolateral beam path. The non-radiopaque PEEK plate allows for assessment of scaffold and cortices. Scale bar = 10 mm.

The rabbits were X-rayed to examine the operated proximal tibia at fixed time points (post-surgery and at weeks 2, 4, 6, 8, 10, 12, 16, 20 and 24; additionally in PR animals at weeks 26, 28 and 30). They were sedated with medetomidine (0.25 mg/kg) and ketamine (15 mg/kg) through intramuscular injection. At the end of the examinations, medetomidine was antagonized with atipamezole (25 mg/kg, intramuscular). Images were acquired at 54.9 kV and 4.5 mAs (Multix Select DR, Siemens GmbH, Erlangen, Germany) in two planes (craniocaudal and mediolateral) (Figure 2). The images were analyzed by three observers using dicomPACS^®^ MobileView software (version 4.0.0.19; Oehm und Rehbein GmbH, Rostock, Germany).

For this purpose, the parameters of scaffold visibility and intactness of the fibula were evaluated descriptively on the radiographs of both levels, and an adapted, modified radiographic union scale for tibial fractures (mRUST) score (Table 2) was used to semi-quantitatively examine the four cortices (cranial, caudal, medial and lateral) of the operated tibia and to assess bone healing after osteotomy [37,38,39]. These scores were then added together to form a total score.

### 2.4. In Vivo µCT Examination

In vivo µCT images (XtremeCT II, Scanco Medical, Zurich, Switzerland) were taken in accordance with the examination interval of the radiological examinations. Sedation and antagonization were performed as for X-rays. In addition, anesthesia was maintained via a laryngeal mask airway (v-gel^®^ rabbit, Docsinnovent Ltd., London, UK) with isoflurane (0.8–1.5 vol% plus oxygen 1.5–2 L/min). The animals were restrained in the supine position with the knee joints extended. The area of the proximal tibia including the implanted wedges, PEEK plate and screws was scanned with the following settings: tube voltage of 68 kV, current of 1470 µA, 1000 projections/180°, integration time of 250 ms and isotropic voxel size of 30.3 µm.

#### 2.4.1. Semi-Quantitative Evaluation of the In Vivo µCT Examination

A scoring system was used for the semi-quantitative evaluation of the µCT scans (Table 3).

**Figure 3:**
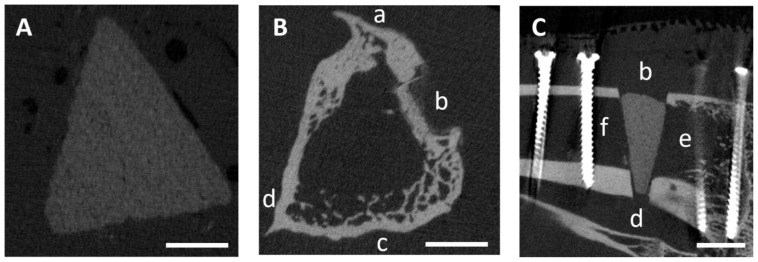
Exemplary (**A**) original µCT scan with scaffold (immediately post-surgery) and (**B**) original µCT scan after 26 weeks (scaffold already degraded); (**C**) reoriented µCT scan (immediately post-surgery). Orientation: (a) cranial, (b) medial (position of the PEEK plate), (c) caudal, (d) lateral, (e) proximal and (f) distal. Scale bar = 5 mm.

The following parameters were examined in the original scan (scaffold and tibia in cross-section) (Figure 3A,B): scaffold visibility, loss of shape and closure of the osteotomy gap by callus tissue. In addition, the scan was reoriented by 90° using the µCT Evaluation Program V6.6 software (Scanco Medical, Zurich, Switzerland) to evaluate the scaffold in the longitudinal section of the tibia. On the reoriented scan (Figure 3C), the parameters of scaffold integration, the resorption zone (the area around the scaffold that is not radiopaque), the delimitable zone within the scaffold, scaffold fit (on the day of surgery), bridging of the medial osteotomy gap (cis-cortex) and the endosteal and periosteal callus formation from proximal and distal positions were examined. Once the scaffold was no longer visible, the remodeling of the trans-cortex was assessed based on its structure.

#### 2.4.2. Quantitative Evaluation of the In Vivo µCT Examination

For quantitative analysis of the scaffolds, volume (scaffold volume, SV) and density (scaffold density, SD) measurements were performed using previously determined material-specific thresholds (Th). Using different gray values of the scaffolds assessed at the time of surgery, the following thresholds were determined independently by three assessors: CMPC Th = 150 and MPC Th = 108. µCT Evaluation Program V6.6 software (Scanco medical, Zurich, Switzerland) was used for the analyses.

### 2.5. Histological Examination and Semi-Quantitative Evaluation

At the end of the respective observation periods, the animals were euthanized after sedation in accordance with animal welfare regulations. For this purpose, propofol (2–4 mg/kg, Narcofol^®^, CP-Pharma GmbH, Burgdorf, Germany) and pentobarbital (200–800 mg/kg, Narkodorm^®^, CP-Pharma GmbH, Burgdorf, Germany) were administered intravenously. The implanted tibiae were then removed and the soft tissue was dissected. The bone–implant composite including the PEEK plate was explanted using a diamond band saw (Cut-grinder, Messner GmbH, Oststeinbek, Germany); for this purpose, the PEEK plate was sawn through above and below the two screws adjacent to the scaffold. The preparation was then carried out as described in Section 2.1.5. Using the cutting–grinding technique according to Donath [34], thick sections (50–80 µm) were prepared by cutting a longitudinal section through the center of the PEEK plate in the mediolateral direction. Subsequently, the most central section from the center of the scaffold was stained with toluidine blue (0.1% toluidine blue O (Waldeck, Münster, Germany)) and then assessed semi-quantitatively with a microscope (Axio Imager Z.2, Carl Zeiss Microscopy GmbH, Oberkochen, Germany) using a scoring system by three people independently of each other (Table 4).

**Figure 4:**
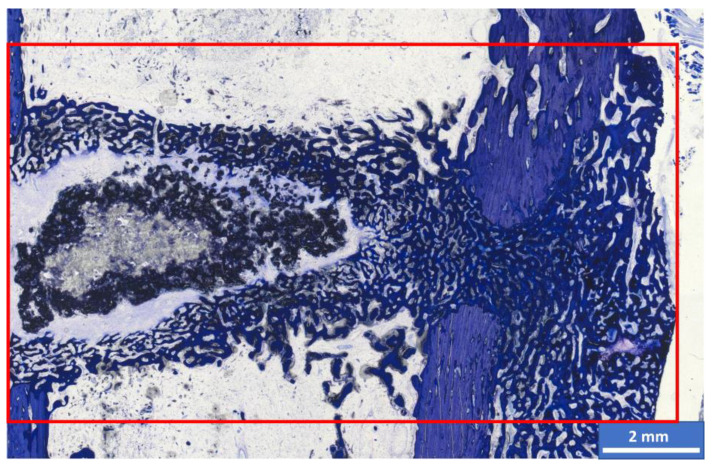
CMPC 6 weeks after surgery: the measuring frame (red rectangle) of the histomorphometric measurements, 3500 × 2000 pixels. Different coloration of the scaffold is visible: in the center of the wedge is a light-colored material area surrounded by a dark-colored scaffold, and a resorption zone is between the wedge and the woven bone.

The bridging of cis- and trans-cortex, remodeling of cis- and trans-cortex (bone maturity), endosteal callus formation proximal and distal to the scaffold, thickness of the trans-cortex (trans-cortex plus periosteal callus), scaffold degradation and integration, and the mean width of the resorption zone (measured at the level of the center of the scaffold) were evaluated at 2.5x magnification. Scaffold areas with different color shades were observed in both materials after 6 weeks (Figure 4). A lighter central area of material was surrounded by dark-colored material. This dark portion was also estimated as a percentage. In addition, the bone–implant composite was examined at the cellular level (occurrence and localization of osteoclasts, osteoblasts, osteoprogenitor cells, adipocytes, bone marrow precursor cells, fibroblasts, fibrocytes, macrophages, foreign body giant cells, lymphocytes, blood vessels (vascularization) and cartilage tissue (magnifications 20x and 40x)). The occurrence of the cell types was analyzed semi-quantitatively in the different localizations of scaffold center, edge, resorption zone and periphery (cis- and trans-cortex; areas proximal and distal to the scaffold). As soon as scaffold degradation was too far advanced, the center (former implantation site based on the screw positions) and trans- and cis-cortex localizations were examined.

#### Histomorphometric Examination

The section was photographed at 2.5x magnification under the microscope using the Zeiss Axio Cam Mrc digital camera and Zeiss ZEN 3.0 software and then analyzed using Zeiss Intellesis software. The percentage area of light and dark scaffold material, woven bone, lamellar bone and cartilage tissue within a defined rectangular measuring frame in the implantation area was measured (Figure 4).

### 2.6. Energy Dispersive X-ray Analysis (EDX) and Scanning Electron Microscopy (SEM) of the Implanted Wedges

Two unstained thick sections (50–80 µm) per material and time group were examined using energy dispersive X-ray spectroscopy (EDX) and scanning electron microscopy (SEM) and prepared analogously to Section 2.1.5. The scaffolds were assessed in the SEM regarding osseointegration based on the surface structure of the thick sections and the contact surface to the surrounding bone. EDX was used to determine the presence of material particles based on the presence of magnesium (Mg) ions or an increased concentration of calcium (Ca) and phosphate (P) ions compared to the surrounding bone.

### 2.7. Statistics

The statistical analysis of the data on the production and characterization of the wedges before surgery was performed using one-way ANOVA analysis and the Tukey test (Origin 7G, OriginLab Corporation, Northampton, MA, USA). Values with *p* < 0.05 were classified as statistically significant. All values are expressed as mean ± standard deviation. The experimental data are based on the following sample numbers: chemical composition (*n* = 3), strength measurements (*n* = 10), porosity measurements (*n* = 3) and SEM/EDX (*n* = 2).

The remaining data collected were analyzed using SPSS Statistics 26.0 (IBM, Armonk, USA) and Microsoft Excel 2019 (Microsoft Corporation, Redmond, WA, USA). Quantitative data were checked for normal distribution using the Shapiro–Wilk test and, in the case of normal distribution, analyzed using the Levene test and then the Welch test or *t*-test. Semi-quantitative data and non-normally distributed data were analyzed using the Mann–Whitney U test. The significance level was set at *p* < 0.01.

## 3. Results

### 3.1. Chemical and Mechanical Properties of the Scaffolds

Post-treatment of the scaffolds with DAHP or phosphoric acid resulted in chemical reactions, which led to the partial conversion of farringtonite to struvite (MPC) (Equation (1)) [40] or of farringtonite and stanfieldite to newberyite (Equations (2) and (3)) and brushite (CMPC) (Equation (3)) [41] (Table 5).

(1) $$2\;{\mathrm{Mg}}_{3}{\left({\mathrm{PO}}_{4}\right)}_{2}+3\;{\left({\mathrm{NH}}_{4}\right)}_{2}{\mathrm{HPO}}_{4}+36{\;H}_{2}0\;\to \;6\;\mathrm{Mg}{\mathrm{NH}}_{4}{\mathrm{PO}}_{4}\;\cdot \;6\;{\;H}_{2}0+H_{3}{\mathrm{PO}}_{4}$$

(2) $${\mathrm{Mg}}_{3}{\left({\mathrm{PO}}_{4}\right)}_{2}+H_{3}{\mathrm{PO}}_{4}+9\;{\;H}_{2}0\;\to \;3\;\mathrm{MgH}{\mathrm{PO}}_{4}\;\cdot \;3\;{\;H}_{2}0$$

(3) $${\mathrm{Ca}}_{4}{\mathrm{Mg}}_{5}{\left({\mathrm{PO}}_{4}\right)}_{6}+3\;H_{3}{\mathrm{PO}}_{4\;}+23\;{\;H}_{2}0\;\to \;4\;\mathrm{CaH}{\mathrm{PO}}_{4}\;\cdot \;2\;{\;H}_{2}0+5\;\mathrm{MgH}{\mathrm{PO}}_{4}\;\cdot \;3\;{\;H}_{2}0$$

The compressive strengths of the scaffolds before implantation differed significantly (*p* < 0.001). They amounted to 21.5 ± 2.8 MPa for the MPC and 15.6 ± 3.4 MPa for the CMPC.

The open porosity also differed significantly between the two groups (*p* = 0.048). For the MPC, it was 9.1 ± 3.6%, while for the CMPC, it was 15.9 ± 2.1%.

### 3.2. SEM/EDX Analyses before Implantation

**Figure 5:**
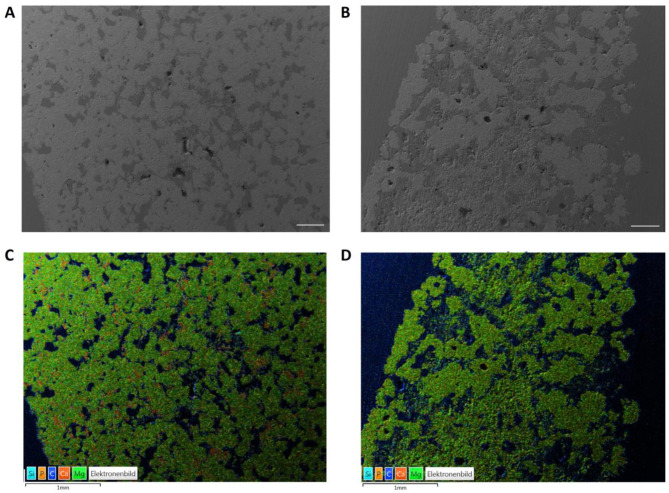
Analyses of the native wedges before implantation. SEM analyses of (**A**) the CMPC and (**B**) the MPC, scale bar = 400 µm. EDX analyses of (**C**) the CMPC and (**D**) the MPC, scale bar = 1 mm.

Compared to the MPC, the CMPC had a more homogeneous structure and consisted mainly of the phases stanfieldite, newberyite and farringtonite (Figure 5A). In the EDX analysis, red parts were visible in the CMPC, which can be assigned to the brushite phase (Figure 5C). The MPC wedge consisted of the two phases farringtonite (coarse-grained) and struvite (smooth and dense). Struvite was predominantly detected in the edge area of the scaffold, whereas farringtonite was visible in the center of the scaffold (Figure 5B,D).

### 3.3. OP and Clinical Examination

The scaffolds could be inserted and fixed almost precisely. All animals showed physiological wound healing and mild to moderate lameness post-operation, which lasted until day 9 in most animals and until day 16 at most in 2/42 animals. In 3/42 animals, due to intermittent lameness, a radiographic examination was performed one week after surgery, which revealed a fibula fracture that did not require treatment. The animals from the PR group showed no lameness after plate explantation and physiological wound healing.

### 3.4. X-ray Examinations

**Figure 6:**
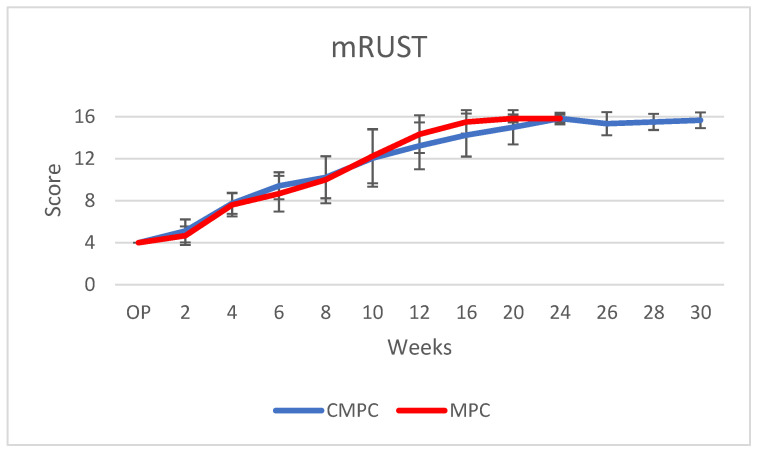
mRUST sum scores of CMPC and MPC from time of surgery to week 30. Plate removal in CMPC at week 24.

Defect healing was similarly fast in both groups. All scaffolds were visible in the radiological examinations from the time of surgery until week 6. The CMPC was no longer visible until week 10 (1/18) at the earliest and week 20 at the latest. The MPC was no longer visible at week 8 (1/12) at the earliest and week 16 at the latest. From week 20 onwards, no implant was radiologically distinguishable from the surrounding bone. In a total of six animals (CMPC, *n* = 5; MPC, *n* = 1), a fracture of the fibula was detected from week 2 onwards, which was covered by callus in the following weeks and ossified after 6 weeks at the latest. The mRUST sum score values were higher with the CMPC than with the MPC from weeks 2 to 8; from weeks 10 to 20, the values of the MPC were higher than those of the CMPC, and in week 24, the values no longer differed (Figure 6). The maximum sum score of 16, which describes radiologically complete osteotomy healing, was reached with both materials at week 10 at the earliest (CMPC, 3/18; MPC, 2/12). From week 16 onwards, the majority of the MPC (5/6) and, from week 20 onwards, the majority of CMPC (8/12) showed complete radiologic osteotomy healing. The mRUST scores of the individual cortices (cranial, caudal, medial and lateral) did not differ significantly between the two materials.

### 3.5. In Vivo µCT Examinations

#### 3.5.1. Semi-Quantitative Evaluation of the Scans

**Figure 7:**
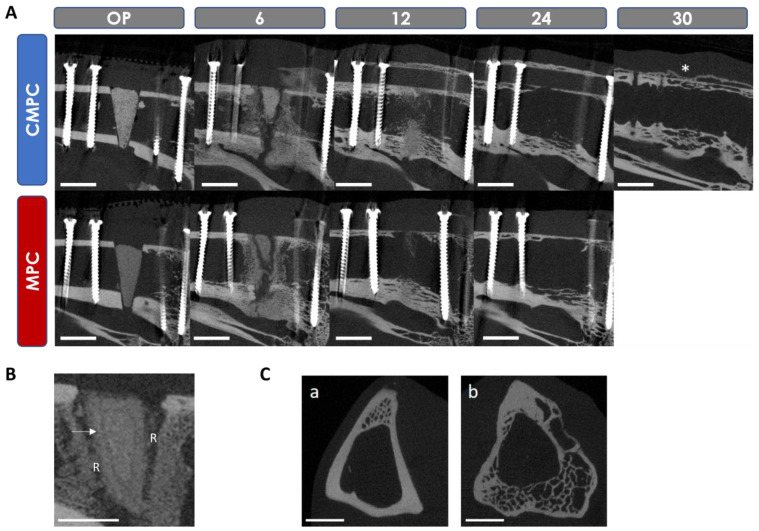
Scaffold degradation of CMPC and MPC after 6, 12 and 24 weeks and of CMPC after 30 weeks in (**A**) reoriented scan. (*) Scan 6 weeks after plate extraction (30 weeks post-operation). (**B**) Arrow: delimitable zone within MPC scaffolds; (R) resorption zone. (**C**) Original scan of (**a**) native compared to (**b**) operated tibia after 30 weeks. Scale bar = 5 mm.

Both materials showed continuous degradation (Figure 7A) and lost shape and visibility from week 2 onwards, with the lateral wedge tip being the first to fragment. From week 8, the wedge shape of all scaffolds was no longer recognizable. At week 20, scaffold remnants could only be seen in one tibia of each group. The presence of a ring-shaped marginal zone within the scaffold, delimited by a line, was only observed in the MPC. This was visible from weeks 2 to 8 and widened over time (Figure 7B). Significantly less scaffold integration was observed with the MPC than with the CMPC in weeks 4, 6 and 8 (*p* ≤ 0.001). From week 2 onwards, a radiolucent zone around the scaffold was noticeable in both materials, which is referred to below as the resorption zone (Figure 7B). In weeks 4, 6 and 8, this zone was significantly more pronounced in the MPC than in the CMPC (*p* ≤ 0.002) and completely surrounded the scaffold. The parameters of scaffold integration and resorption zone could no longer be assessed from week 12 due to the advanced degradation of most of the scaffolds. The formation of endosteal callus was observed in both groups from week 2 onwards. This formed from the tibial endosteum and was confluent in the center of the medullary cavity. In the CMPC, it was most pronounced between weeks 4 and 6, in the MPC, it was from weeks 4 to 8 and was degraded again after 10 weeks at the earliest and 30 weeks at the latest. In weeks 10 and 12, this growth differed significantly between the MPC and the CMPC (*p* = 0.005). At week 10, the CMPC showed an interconnected callus in 10/18 animals (connecting the medial and lateral endosteum) and the MPC showed a non-connected callus in 7/12 animals. At week 12, the endosteal callus was still visible in 14/18 CMPC scaffolds, which was already degraded in the majority of animals with the MPC. The callus formations assessed proximal and distal to the scaffold were symmetrical in most animals, with three tibiae (MPC, *n* = 2; CMPC, *n* = 1) showing more callus distally than proximally. Periosteal callus at the trans-cortex was observed in all scaffolds from week 2 until the end of the observation period. It was most pronounced in the CMPC from weeks 4 to 8 and in the MPC in week 6 and became flatter again over time. The original shape of the tibia had not been restored by the end of the observation periods. After restoration of the bone marrow cavity, the trans-cortex showed a widened, cancellous and loosened structure in all animals compared to the original cortex (Figure 7C).

#### 3.5.2. Quantitative Evaluation of the Scans

The volume and density measurements could only be carried out up to week 16 at the latest, as most of the scaffolds were no longer visible in the in vivo µCT or could no longer be clearly distinguished from the bone. A continuous decrease in volume was observed for both materials. The scaffolds showed a volume decrease of 70.84% (CMPC) and 72.44% (MPC) after 6 weeks and a loss of 97.63% (CMPC) and 99.87% (MPC) after 16 weeks. The density of both materials decreased slightly over the observation period. The decrease in density after 16 weeks was 1.83% for the CMPC and 19.61% for the MPC.

### 3.6. Histological Examination

#### 3.6.1. Semi-Quantitative Assessment

The CMPC showed complete bridging of the cis-cortex (the medial side under the PEEK plate) earlier compared to the MPC. After 6 weeks, the cis-cortex was 1–25% bridged in 3/6 scaffolds of both groups. One scaffold of the CMPC group already showed an almost complete bridging of 76–100%. One scaffold from each group showed no bridging of the cis-cortex at this time. After 12 weeks, the cis-cortex was completely bridged in all CMPC scaffolds and in the majority of MPC scaffolds (66.7%). One MPC scaffold showed no bridging at this time. After 24 weeks, complete bridging of the cis-cortex was observed in all scaffolds.

The trans-cortex (the lateral side at the tip of the wedge) was 76–100% bridged in most animals for both materials (CMPC, *n* = 5; MPC, *n* = 4) after 6 weeks. After 12 weeks, the trans-cortex was bridged in all animals.

In the majority of scaffolds in both groups (CMPC, *n* = 5; MPC, *n* = 4), remodeled woven bone with no to little lamellar bone was observed at the cis-cortex after 6 weeks. In one MPC implant, woven bone with cartilage tissue was observed at the cis-cortex. No remodeling was observed in one animal of each group at this time. After 12 weeks, most scaffolds (5/6 each) showed woven bone with little to no lamellar bone. The cis-cortex of one CMPC animal was almost completely remodeled into lamellar bone. In one implant of the MPC group, there was no remodeling at this time. After 24 weeks, the cis-cortices of all scaffolds were almost completely remodeled into lamellar bone.

After 6 weeks, the trans-cortex of 50% of the CMPC group was remodeled into woven bone with little to no lamellar bone. The trans-cortices of the other 50% (CMPC) and all MPC animals consisted of woven bone with cartilage tissue. After 12 weeks, the trans-cortex of two CMPC scaffolds and one MPC implant consisted mainly of lamellar bone. The majority of the MPC scaffolds and two CMPC scaffolds showed woven and lamellar bone and two other animals (CMPC) showed woven bone with cartilage tissue. After 24 weeks, all trans-cortices were almost completely remodeled into lamellar bone.

**Figure 8:**
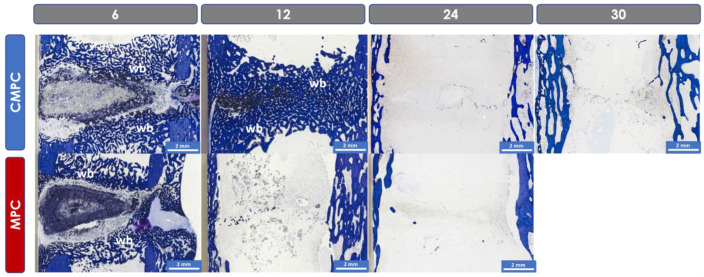
Histological thick sections (toluidine blue; magnification ×2.5/0.085) of the CMPC and the MPC after 6, 12 and 24 weeks and of the CMPC after 30 weeks. Blue: bone tissue. After 6 weeks, all scaffolds show a lighter-colored central area with an adjacent darker-colored zone. A cell-rich resorption zone is visible around the scaffolds. (wb) Woven bone is visible after 6 weeks in both materials, and after 12 weeks, it has already regressed in the MPC group.

An endosteal callus (woven bone) was observed in both groups, which was built up or decreased again over time (Figure 8). This process took place somewhat faster in the MPC group than in the CMPC group. After 6 weeks, the endosteal callus was 76–100% continuous (confluent from the medial and lateral tibial endosteum) in almost all but 2/6 (MPC) animals. In these two animals, a more distal than proximal endosteal callus was observed. After 12 weeks, half of the CMPC group still had a continuous callus, whereas in the majority of the MPC group, it had already regressed. In both materials, the callus was no longer visible after 12 weeks in a small number of animals (CMPC, *n* = 2; MPC, *n* = 1). After 24 weeks, the endosteal callus was completely decreased in all tibiae.

The mean thickness of the trans-cortex (measured including the periosteal callus) decreased continuously over time in both groups (6 weeks: CMPC: 3.97 mm and MPC: 4.00 mm; 12 weeks: CMPC: 3.23 mm and MPC: 3.77 mm; 24 weeks: CMPC: 3.13 mm and MPC: 2.53 mm; 30 weeks: CMPC after plate removal: 3.07 mm).

All scaffolds showed continuous scaffold degradation from the outside in, with CMPC scaffolds degrading slightly earlier than MPC ones. After 6 weeks, almost all scaffolds of the MPC group (4/6) and half of the CMPC group were degraded to 26–50% and two scaffolds each were degraded to 51–75%. After 12 weeks, all scaffolds made of CMPC and the majority of the MPC group (4/6) were degraded to 76–100%, as well as 2/6 MPC scaffolds being degraded to 51–75%. After 24 weeks, almost complete degradation was observed in all scaffolds.

CMPC scaffolds showed slightly better scaffold integration. Most scaffolds in both groups (CMPC, *n* = 5; MPC, *n* = 4) were 1–25% integrated into new bone tissue after 6 weeks. After 12 weeks, half of the CMPC scaffolds showed 76–100% scaffold integration, whereas 4/6 of the MPC scaffolds showed no integration. After 24 weeks, scaffold degradation was so advanced that only isolated material particles could be detected, which were completely embedded in bone tissue.

In all scaffolds, a cell-rich resorption zone was observed around the scaffold after 6 weeks. This zone contained osteoprogenitor cells, fibroblasts, fibrocytes, macrophages, foreign body giant cells, lymphocytes and blood vessels. The zone became narrower over time as new bone tissue was formed from the periphery toward the scaffold and it was no longer present after 24 weeks at the latest. The MPC showed a wider resorption zone compared to the CMPC at all time points (6 weeks: CMPC: 1.12 mm and MPC: 1.3 mm; 12 weeks: CMPC: 0.35 mm and MPC: 1.05 mm).

A different coloration of the scaffold material was observed over time. At week 6, all scaffolds consisted of a central, light gray-to-purple portion and an outer, dark purple-to-black portion (Figure 4 and Figure 8). The groups showed a different proportion of dark material in the outer edge area after 6 weeks. It amounted to 27% in the CMPC group and 62% in the MPC group. After 12 weeks, only diffusely distributed dark material particles could be observed in the former scaffold area.

**Figure 9:**
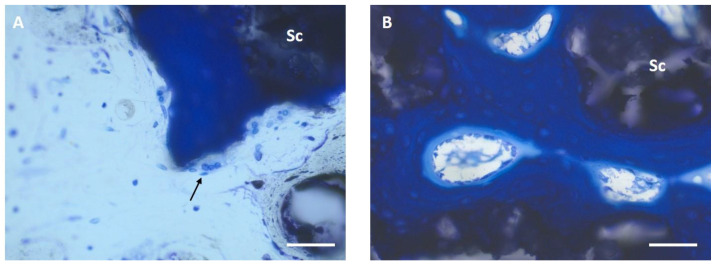
Histologic thick sections of the CMPC after 12 weeks (toluidine blue; ×40/0.75). (**A**) Osteoclast-like cells (arrow) on woven bone (dark blue), which is directly adjacent to the scaffold (Sc). (**B**) Osteoid margin with osteoblasts. Scale bar = 50 µm.

Multinucleated osteoclast-like cells were observed in both groups at all time points, mainly in the periphery of new bone tissue and, in the CMPC, also occasionally on the surface of the marginal cement particles when new bone was formed on them (Figure 9A).

Woven bone, which grew from the periphery toward the scaffold, showed osteoid seams with osteoblasts in both groups in the vicinity of the scaffold. After 12 weeks, osteoblasts were also found on the scaffold material with direct bone contacts (Figure 9B). These scaffold–bone contact sites were observed more frequently in the CMPC group than in the MPC group.

Osteoprogenitor cells were only seen up to week 12. These cells were observed sporadically at the scaffold edges, but mainly in large quantities within the resorption zone.

In weeks 6 and 12, many fibroblasts and fibrocytes were seen in the resorption zone. In the MPC, they were also observed at the edge of the material in week 6, and from week 12 onwards, they were also in the marginal areas of the CMPC.

Adipocytes and bone marrow precursor cells were only detected after 12 weeks. After 24 weeks, the medullary cavity was filled with physiological bone marrow in all animals.

Macrophages were observed in all examined areas except in the scaffold center. They were present at weeks 6 and 12 mainly on and between the marginal material particles and in the resorption zone and were still detectable on material residues after 24 weeks in the MPC group and after 30 weeks in the CMPC group.

Foreign body giant cells were observed in weeks 6 and 12 in both groups directly on the scaffold material and in the resorption zone.

Lymphocytes were mainly present in the resorption zone and between the marginal particles at weeks 6 and 12, and at week 12, they were observed sporadically only in the MPC group.

Blood vessels were present in both materials at every observation point. They could be seen between the cement particles, especially at the transition from the scaffold center to the edge (CMPC), in the resorption zone and in the periphery between the bone trabeculae, but not within the scaffold center. After 6 weeks, more blood vessels were detected within the CMPC scaffolds than in the MPC ones.

Cartilage tissue was formed in both groups, especially at the trans-cortex, and was seen more frequently in MPC animals after 6 weeks. At this point, cartilage tissue was seen in all MPC animals and in three CMPC scaffolds at the trans-cortex. After 12 weeks, it was seen only sporadically at the cortex in both groups.

#### 3.6.2. Histomorphometric Examinations

The proportion of scaffold material was dark or light and decreased continuously within the measurement frame for both groups. After 6 weeks, the CMPC scaffolds had a higher proportion (3.2%) of light-colored material than the MPC ones (1.9%). The proportion of dark material was always higher in the MPC scaffolds than in the CMPC ones. In both materials, the formation of woven bone was observed, which was continuously remodeled into lamellar bone. More woven bone was always formed in the CMPC than in the MPC. After 6 weeks, the CMPC had a significantly higher proportion of woven bone (31.5%) compared to the MPC (21.3%) (*p* = 0.004). New woven bone was also observed in the CMPC after 30 weeks, which formed on the cis-cortex as a result of the plate removal. Similar amounts of cartilage tissue could be measured in both groups, which decreased over time. There was a greater difference after 6 weeks where the MPC showed a higher proportion of cartilage tissue (1.4%) compared to the CMPC (0.5%).

### 3.7. SEM/EDX Analyses after Implantation

Continuous scaffold degradation was observed in both groups.

**Figure 10:**
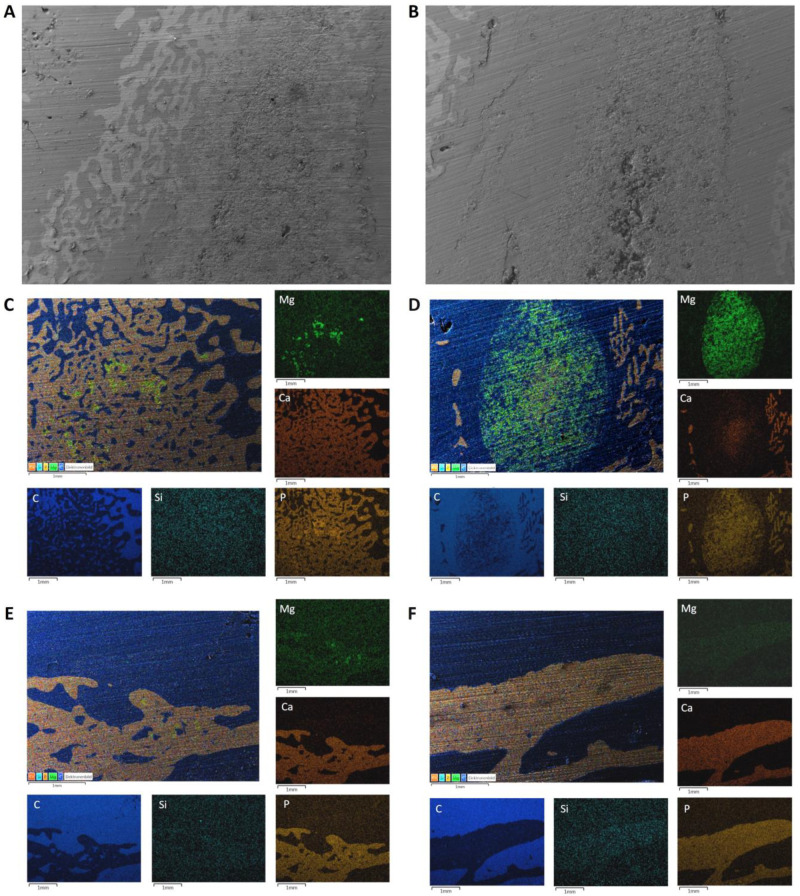
SEM and EDX analyses of the histological thick sections. SEM analyses of (**A**) the CMPC after 6 weeks and (**B**) the MPC after 6 weeks. EDX analyses of (**C**) the CMPC after 12 weeks, (**D**) the MPC after 12 weeks, (**E**) the CMPC after 30 weeks and (**F**) the MPC after 24 weeks. Scale bar = 1 mm.

After 6 weeks, most of the scaffolds were still present. In the marginal area of the CMPC, woven bone was growing onto and into the scaffold (Figure 10A). In the MPC, the scaffolds were clearly separated from the woven bone by a resorption zone, which meant that no bone had grown onto the scaffold. However, scaffold particles (Mg) were present in this zone. In the middle of the MPC scaffolds was an area consisting of farringtonite that had not been reacted with DAHP (Figure 10B).

After 12 weeks, CMPC remnants (roughened areas) were still visible and well integrated into the trabecular structure (Figure 10C). Multiple new bone trabeculae had formed in the scaffold area. MPC scaffolds were also further degraded but still surrounded by a resorption zone. The Ca distribution in the EDX measurements shows that new bone or precipitated hydroxyapatite formed in the implant (Figure 10D).

After 24 weeks, only a very small area of scaffold material was still present in a CMPC specimen. This was seen in the bone marrow not embedded in a trabecular structure. New bone had formed at the edge of the implant. The other CMPC sample had no residual material and new trabeculae had formed, filling the implantation area. No more material was visible in the MPC group. A narrow strip of bone was present at the edges of the former scaffold area and the center of the area was filled with bone marrow (Figure 10F).

In one of the CMPC samples, a few scaffold particles were still present in the newly formed trabeculae after 30 weeks. At the edge of the scaffold area, there was new bone with material remnants (Figure 10E). In addition, a large area of bone marrow was present in the center. In the other specimen of this group, no more material was detectable. No bone trabeculae were visible in the implantation area. A thin bone seam was visible at the implantation margin.

Measured silicon comes from the glass substrate and is therefore negligible.

## 4. Discussion

CMPCs and MPCs are superior to CPCs due to their improved mechanical properties and higher in vivo solubility [11,42,43,44]. Therefore, they are becoming increasingly important for their use as bone substitutes and have already been successfully tested in unloaded defect models [13,23,24,43]. In the present study, wedge-shaped MPC- and CMPC-based scaffolds were examined for the first time in the partially loaded, segmental defect model in the rabbit tibia. The scaffolds were 3D powder-printed, as this method is well suited to produce dimensionally stable, defect-specific bone substitutes with a customizable macrostructure [29,45,46,47]. Therefore, they are particularly suitable for larger or more complex defects. Another characteristic of powder-printed materials is the bone-like, interconnecting microporosity of up to 70%, which promotes nutrient diffusion, vascularization and thus osteogenesis [27,30,48]. The materials used were post-treated with DAHP (MPC) and phosphoric acid (CMPC) to increase the final strength [28] and were examined with regard to their in vivo degradation and osseointegration behavior. The animal model and the surgical method used have already been established, and the present study was adapted from a study by Schmidt et al. [35].

In terms of compressive strength and open porosity, the two materials used in the present study differed significantly from each other. The MPC exhibited both a higher compressive strength and a lower open porosity compared to the CMPC before implantation. This inverse correlation of porosity and compressive strength has already been established in other publications [49,50]. It is assumed that the strength development (cement hydration) takes place in two stages. First, the maximum pore size determines the strength (pore size-controlled stage). If this pore size falls below a critical defect size, the porosity determines the modulus of elasticity and thus the strength of the cement (porosity-controlled stage) [51].

In the present study, minimal dislocation of the wedge was observed over time in 3/42 animals (MPC, *n* = 2; CMPC, *n* = 1), as an asymmetric callus distribution (distal > proximal) was seen in both the µCT and histologic examinations. Since no other abnormalities were observed in these animals, it is assumed that loosening of the defect fixation occurred as a result of post-operative stress and that the scaffolds therefore slipped out of place.

As in previous in vivo studies with MPCs and CMPCs, all animals had physiological wound healing [20,24] and showed lameness, as expected for an invasive surgical method such as the one used here.

The visibility of the scaffolds in the radiologic analyses was similar in both groups and decreased over time, indicating progressive degradation. CMPC scaffolds were visible longer than MPC scaffolds, indicating faster degradation of the MPC. However, the prolonged visibility could also be attributed to the calcium (Ca) contained in the CMPC. Past studies have already shown that CPCs exhibit stronger and longer X-ray visibility compared to MPCs and CMPCs due to their increased Ca content and slower degradation rate [16,24,52]. Since none of the scaffolds could be distinguished from the surrounding bone on the X-ray images in the present study after 20 weeks, it can be assumed that the scaffolds were already largely degraded at this time. This was also confirmed in the in vivo µCT measurements, as at this time, only one animal of each group could be found with material remains.

Fibula fractures, which were detected radiographically in 6/42 animals in the first two weeks after surgery, had occurred in both material types because of surgical drilling of the fibula and therefore were considered independent of the material. No effects on subsequent scaffold degradation and bone healing of the defects could be determined.

Both materials showed a similar osteotomy healing process over the observation period. Radiologically complete defect healing, which is achieved with a maximum mRUST sum score (no osteotomy line visible on all four cortices and the osteotomy gap completely bridged with the callus or remodeled), occurred in both groups from week 16 onwards. The assessment of defect healing using radiographs by evaluating callus formation and the visibility of a fracture line at the four cortices (RUST or mRUST score) has already been successfully applied to tibial and femoral shaft fractures in humans and in animal models [37,38,39,53,54].

In the present study, a radiolucent resorption zone around the scaffold was noticeable from week 2 in the X-ray and µ-CT analyses for both materials. This zone was more pronounced in the MPC than in the CMPC at weeks 4, 6 and 8 and correlated with the simultaneously lower scaffold integration, i.e., fewer visible scaffold–bone contact points, of the MPC compared to the CMPC. The better scaffold integration of the CMPC compared to the MPC observed in the µCT examination could also be verified histologically. While both groups showed similar scaffold integration behavior at week 6, they differed significantly at week 12, at which time half of the CMPC group showed almost complete scaffold integration into new bone tissue, whereas almost all MPC scaffolds were still not integrated into the bone due to complete degradation or the presence of a resorption zone. This lack of scaffold–bone contact probably results from the faster degradation of the MPC compared to the CMPC, as although woven bone was formed around the implant, the bone growth rate did not match the scaffold degradation rate. The resorption zone could no longer be clearly assessed by in vivo µCT from week 12 onwards, as scaffold degradation had progressed too far. Histological examinations verified this zone as a cell-rich resorption zone, which contained fibrovascular cells and macrophages. Resorption zones are already known in the literature and correlate with an accelerated degradation rate [20,24]. Defect fixation was stable over the observation period despite the occurrence of a resorption zone.

In the present study, a faster degradation rate of the MPC was observed compared to the CMPC. A possible explanation for this is provided by Kowalewicz et al., who investigated cylindrical scaffolds made of CMPC in vivo and discussed in their study, among other things, the relationship between the chemical solubility and the influence of the wt% proportion of the binder phases farringtonite, struvite, brushite and newberyite on the degradation rate of the scaffolds. The chemical solubility of newberyite is the highest, followed by brushite and then struvite [11,24]. In the study by Kowalewicz et al., the CMPC scaffolds were post-treated with DAHP or phosphoric acid in a manner equivalent to the present study, resulting in the same binder phases (post-treatment and resulting binder phase: DAHP -> struvite; phosphoric acid -> newberyite and brushite) [19,47,52]. These rapidly soluble binder phases hold the poorly soluble phases farringtonite and stanfieldite together. In the present study, a low proportion of rapidly soluble struvite (19 wt%) was present in the MPC compared to the high proportion of newberyite (58 wt%) in the CMPC. This could therefore have resulted in a faster degradation of the MPC, as the low proportion of the binder phase struvite was possibly dissolved more quickly and the scaffolds were therefore no longer sufficiently held together. The dissolution of the even more rapidly soluble binder phases newberyite and brushite took longer due to their higher content and degradation was correspondingly slower. A similar observation was also described in an in vitro study by Gefel et al. [47].

In the present study, the degradation of both materials had already progressed so far after 24 weeks that histologically, only isolated scaffold particles could be detected, which were completely integrated into bone tissue. Kowalewicz et al. [24] also observed in their in vivo study on CMPCs that they were almost completely degraded after 24 weeks.

In both groups, the formation of the endosteal and periosteal callus (trans-cortex) was observed from week 2 onwards, which built up and decreased over time. Schmidt et al. [35] used the same defect model in rabbits as in the present study, but with a scaffold made of a magnesium alloy, and also observed these callus formations, which occur physiologically as part of secondary fracture healing [55,56]. The scaffold fixation system used in the present study (the PEEK plate and screws) may have enabled interfragmentary movements that stimulated callus growth and thus bone healing, as has already been described in the literature for similar systems [55]. At weeks 10 and 12, CMPCs showed more pronounced endosteal callus formation compared to MPCs, which could be due to the high efficiency of CMPCs to form new bone. This was also confirmed in a study by Wu et al. [21] in which cylindrical CMPC and CPC scaffolds were implanted into drilled hole defects in rabbits. More newly formed bone was measured in the defect localization with CMPCs compared to CPCs. Zeng et al. [42] investigated granules of MPCs, CPCs and CMPCs and their effects on the cell proliferation and osteogenic cell differentiation of mesenchymal stem cells and on in vivo osteogenesis during maxillary sinus floor surgery in rabbits. It was found that CMPCs showed enhanced cell proliferation and differentiation compared to MPCs and induced increased bone growth in vivo compared to pure CPCs and MPCs.

The reduction in the endosteal callus, the flattening of the periosteal callus, the decrease in the mean thickness of the trans-cortex over time and the restoration of the bone marrow cavity observed in the present study occurred in the context of physiological bone remodeling, as also described in the literature [55].

The quantitative evaluation of the volume and density of both materials using µCT measurements could only be carried out up to week 16, as almost all scaffolds were no longer visible after this time or could no longer be clearly distinguished from the surrounding bone due to a similar radiographic density. A similar problem has already been identified in other studies [24,57]. In the present study, both materials showed a continuous decrease in volume, which resulted from a continuous scaffold degradation of both groups. The MPC showed a slightly greater loss of volume and density compared to the CMPC. This observation confirms the hypothesis that MPCs exhibit faster degradation than CMPCs, as already suggested by the results from the semi-quantitative µCT studies. The faster bridging on the medial side (under the PEEK plate) in CMPC scaffolds compared to MPCs may have occurred due to the potentially higher osteogenic efficiency of CMPCs compared to MPCs and due to the narrower resorption zone in CMPCs, as described above. The earlier bridging of the trans-cortex (lateral) compared to the cis-cortex is due, on the one hand, to the greater distance between the defect ends at the cis-cortex, and, on the other hand, to the fact that the formation of new bone directly under the plate was mechanically disturbed or could not be stimulated by interfragmentary movements [55,58]. The plate fixation partially relieved the cortical bone at this point, reducing the mechanical stimulus for new bone formation. This phenomenon is referred to as “stress shielding” and ultimately leads to a local decrease in bone tissue [59].

In general, the formation of cartilage tissue can be considered physiological, as it occurs during secondary fracture healing as part of endochondral ossification [55,60]. However, the higher and more frequent cartilage content in MPCs compared to CMPCs after 6 weeks, which could also be confirmed histomorphometrically, could also be related to the faster degradation of MPCs. Due to the rapid degradation of MPC scaffolds, the defect loses a certain degree of mechanical stability, which can lead to an increase in minimal movement, and this leads to the bone trying to stabilize the defect through excessive callus formation [60,61].

Biomechanical stress has a major influence on defect healing. The stimulation of the cells in the callus determines the quality of the callus tissue formed. Too little stimulation results in non-union, whereas too much stimulation leads to pure fibrocartilage formation (pseudarthrosis). With ideal defect mobility and an appropriate gap width, suitable cartilage is formed, which can then be remodeled [58,62]. In the present study, it was observed that the callus formed in both groups was remodeled into bone tissue over time and always ensured mechanical stability. Histologically, a continuous, centripetal scaffold degradation was also observed in all scaffolds, whereby a slightly faster degradation rate of the CMPCs compared to the MPCs was observed in these examinations up to week 12.

The delimitable marginal zone of MPCs observed in the µ-CT, histological and SEM examinations was probably struvite, which was formed as a result of the reaction of farringtonite with DAHP during the production and post-treatment of the wedges. Using SEM, the present study showed that there was a zone in the middle of the MPC scaffold that consisted of farringtonite which had not reacted with DAHP. As no struvite was formed there, it can be assumed that a lower compressive strength was present at this site than in the rest of the scaffold. Non-reacted cement powder in the scaffold center of the CMPC cylinders post-treated with DAHP was also observed by Kowalewicz et al. [24]. The increase in compressive strength of MCPs due to post-treatment with DAHP has also been reported by other research groups [31,63].

In the histological analysis, the detected cells were mainly seen at and between the marginal particles of the scaffolds and the growing bone tissue, and in the resorption zone. The cell types that appeared are considered a physiological response [20,24,64]. During centripetal scaffold degradation, marginal cement particles were presumably the first to detach, thus allowing cells to penetrate.

The PEEK plate was removed to investigate the further remodeling of the bone after defect healing and scaffold degradation. The CMPC showed a slightly slower degradation rate in the early weeks after surgery, as well as better scaffold integration compared to the MPC. To reduce the number of test animals used, only CMPC implants were therefore used for the plate extraction part of the study. After explantation of the PEEK plates, all tibiae were stable and bone remodeling progressed. This could be verified histologically, as the newly formed woven bone was remodeled into lamellar bone in all animals as part of endochondral ossification and the medullary cavity was filled with physiological bone marrow again after 24 weeks at the latest. The diameters of the trans-cortices decreased over time and also after the removal of the plate and approached the initial tibial shape. In the µ-CT examinations, it was observed that the original shape of the tibia had not yet been restored after 30 weeks, as the cortices still showed a widened, loosened structure. According to the literature, this course of fracture healing is considered physiological [55,61].

## 5. Conclusions

The 3D powder-printed, wedge-shaped scaffolds made of CMPC and MPC used in the partially loaded defect model in the present study showed excellent biocompatibility, osteoconductivity and continuous scaffold degradation. After 24 weeks at the latest, both scaffolds were almost completely degraded, resulting in a restoration of the medullary cavity. The cortices were largely remodeled into lamellar bone so that a removal of the PEEK plate was possible. Even after plate removal, there were no complications and the bone continued to remodel. In all tibiae of both groups, the defect remained mechanically stable. However, especially in the early weeks, the CMPC showed a somewhat slower degradation rate which corresponded more to the growth rate of the bone compared to the MPC. The fact that a central, presumably more unstable zone of unreacted farringtonite was detected in MPC scaffolds in the SEM measurements indicates that the manufacturing and post-treatment process should be revised and optimized further to ensure the production of scaffolds that are as uniform as possible. CMPC scaffolds showed promising results in the present study and should be further analyzed, for example in defect models with higher loads or in defects of larger dimensions, also with a stronger focus on biomechanical properties.

## Figures and Tables

**Table 1:** Chemical composition of cement raw powder mixtures in mol.

Ca_x_Mg_3−x_(PO_4_)_2_	CaHPO_4_	CaCO_3_	MgHPO_4_ 3H_2_O	Mg(OH)_2_
x = 0.00	-	-	2.00	1.00
x = 0.75	0.50	0.25	1.50	0.75

**Table 2:** Scoresheet for X-ray examination.

mRUST Score	Osteotomy Line	Callus
1	Visible	Missing
2	Visible	Available
3	Visible	Bridged
4	Not visible	Completely bridged or remodeled

**Table 3:** Scoresheet for semi-quantitative µ-CT examinations.

Parameters	Score 0	Score 1	Score 2
Scaffold visibility	Not visible	Partially visible	Fully visible
Loss of shape	Wedge shape no longer recognizable	Wedge shape partially recognizable	Wedge shape clearly recognizable
Closure of osteotomy gap by callus tissue	Scaffold completely covered by callus	Partial callus formation	No callus formation
Scaffold integration	Continuous visible contact surface between scaffold and bone	Contact surface between scaffold and bone partially interrupted	No visible scaffold–bone contact
Resorption zone	No resorption zone	Scaffold partially surrounded by resorption zone	Scaffold completely surrounded by resorption zone
Delimitable zone within scaffold	No zone delimitable	Zone indistinctly delimitable	Zone clearly delimitable
Scaffold fit (on day of surgery)	Scaffold is attached to the cortices	Scaffold does not lie medially or laterally against cortical bone	Scaffold is neither medial nor lateral to cortical bone
Bridging of medial osteotomy gap (cis-cortex)	Complete bridging	Partial bridging	No bridging
Endosteal callus formation proximal to scaffold	None	Minor	Medium to high
Endosteal callus formation distal to scaffold	None	Minor	Medium to high
Periosteal callus formation (trans-cortex)	None	Minor	Medium to high
Remodeling trans-cortex	Even and narrow = physiological	Loosened and cancellous	Heavily curved

**Table 4:** Scoresheet for semi-quantitative histology.

Parameters	Score 0	Score 1	Score 2	Score 3	Score 4
Cis-cortex bridging	76–100%	51–75%	26–50%	1–25%	Not bridged
Trans-cortex bridging	76–100%	51–75%	26–50%	1–25%	Not bridged
Cis-cortex remodeling (bone maturation)	Mainly lamellar bone	Woven bone with no to little lamellar bone	Woven bone with cartilage	Cartilage tissue	No remodeling
Trans-cortex remodeling (bone maturation)	Mainly lamellar bone	Woven bone with no to little lamellar bone	Woven bone with cartilage	Cartilage tissue	No remodeling
Proximal endosteal callus	76–100%	51–75%	26–50%	1–25%	No callus
Distal endosteal callus	76–100%	51–75%	26–50%	1–25%	No callus
Scaffold degradation	76–100%	51–75%	26–50%	1–25%	No degradation
Scaffold integration	76–100%	51–75%	26–50%	1–25%	No integration
Resorption zone	Measured at level of scaffold center in mm				
Proportion of dark-colored material	Estimated percentage of scaffold				
Thickness of the trans-cortex in mm	Measured including periosteal callus				

**Table 5:** Chemical composition of scaffolds in wt%.

	Brushite	Stanfieldite	Farringtonite	Struvite	Newberyite	Periclase
MPC	-	-	81.0 ± 3.2	19.0 ± 3.2	-	-
CMPC	10.9 ± 2.4	20.1 ± 4.9	10.3 ± 1.4	-	58.1 ± 3.9	0.6 ± 0.1

## Data Availability

The data presented in this study are available on request from the corresponding author.

## Abbreviations

µ-CT	µ-computed tomography
ATMP	Amorphous trimagnesium phosphate
Ca	Calcium
CMPC	Ca_0.75_Mg_2.25_(PO_4_)_2_ post-treated with phosphoric acid
CMPCs	Calcium magnesium phosphate cements
CPCs	Calcium phosphate cements
CTMP	Crystalline trimagnesium phosphate
DAHP	Diammonium hydrogen phosphate
EDX	Energy dispersive X-ray analysis
HA	Hydroxyapatite
Mg	Magnesium
MPC	Mg_3_(PO_4_)_2_ post-treated with diammonium hydrogen phosphate
MPCs	Magnesium phosphate cements
mRUST	Modified radiographic union scale for tibial fractures
P	Phosphate
PA	Phosphoric acid, H_3_PO_4_
PEEK	Polyetheretherketone
PR	Plate removal
RT	Room temperature
SD	Scaffold density
SEM	Scanning electron microscopy
SV	Scaffold volume
TCP	Tricalcium phosphate
Th	Threshold
